# Designing Efficient Processes for Sustainable Bioethanol and Bio-Hydrogen Production from Grass Lawn Waste

**DOI:** 10.3390/molecules25122889

**Published:** 2020-06-23

**Authors:** Georgia Antonopoulou

**Affiliations:** Institute of Chemical Engineering Sciences, Stadiou, Platani, 26504 Patras, Greece; geogant@chemeng.upatras.gr; Tel.: +30-261-096-5318

**Keywords:** pretreatment, fermentation, hydrolysis, grass lawn, ethanol, hydrogen, whole slurry, separation, process scheme

## Abstract

The effect of thermal, acid and alkali pretreatment methods on biological hydrogen (BHP) and bioethanol production (BP) from grass lawn (GL) waste was investigated, under different process schemes. BHP from the whole pretreatment slurry of GL was performed through mixed microbial cultures in simultaneous saccharification and fermentation (SSF) mode, while BP was carried out through the C5yeast *Pichia stipitis*, in SSF mode. From these experiments, the best pretreatment conditions were determined and the efficiencies for each process were assessed and compared, when using either the whole pretreatment slurry or the separated fractions (solid and liquid), the separate hydrolysis and fermentation (SHF) or SSF mode, and especially for BP, the use of other yeasts such as *Pachysolen tannophilus* or *Saccharomyces cerevisiae*. The experimental results showed that pretreatment with 10 gH_2_SO_4_/100 g total solids (TS) was the optimum for both BHP and BP. Separation of solid and liquid pretreated fractions led to the highest BHP (270.1 mL H_2_/g TS, corresponding to 3.4 MJ/kg TS) and also BP (108.8 mg ethanol/g TS, corresponding to 2.9 MJ/kg TS) yields. The latter was achieved by using *P. stipitis* for the fermentation of the hydrolysate and *S. serevisiae* for the solid fraction fermentation, at SSF.

## 1. Introduction

Bioeconomy holds a very important place in an overall circular economy concept. Renewable resources from land and sea, such as wastes and residues of human activities, are considered a source of valuable molecules and fuels, thus, tackling environmental and economic issues. In recent years, many strategies and policies have been adopted across the world to enhance the circular economy model and to promote the production and consumption of biofuels as alternatives to fossil fuels, with potential benefits including the reduction of greenhouse gas emissions, independence from natural resources and security of the food chain [1]. Biohydrogen and bioethanol are promising CO_2_ neutral biofuels, which are nontoxic and may be derived from a wide variety of raw, renewable materials [2].

Production of biofuels such as biohydrogen or bioethanol from energy crops rich in sugars or starch, such as corn, sweet sorghum [3,4] or wheat [5], has a low contribution to the total fuel demand, with negative environmental impact [1], competing also with food and feed supply. As a result, the valorization of wastes or plant/crop-residues, rich in lignocellulose, towards second generation biofuels seems to be the sole alternative [6,7]. However, their commercial production has not developed yet, being limited to pilot-scale plants in North America, Europe and a few emerging countries (e.g., Brazil, China, India and Thailand) [8]. This is the case mainly because of the higher investment cost compared to fossil fuels or first-generation biofuels. To reduce the cost and recover the limiting factors for the commercialization of second generation ethanol or hydrogen, efforts should be paid in developing both effective and low-cost pretreatment technologies [9] and in exploring zero-cost feedstocks, such as potato peels [6], kitchen wastes [7], expired rice [10] or grass lawn (GL) waste [11], which contain a high content of carbohydrates.

GL is an abundant carbon source with increased production potential due to the recent trends for green cities with large green areas, green campuses and gardens of urban houses. GL waste remains to a high extent unexploitable, since it is usually burned, discarded or disposed in landfills [11,12], being collected together with the solid municipal wastes, thus causing environmental problems [13]. Its abundance, together with the possibility of exploiting the entire biomass, render GL waste an alternative promising biomass for second generation biofuels such as hydrogen or bioethanol [14]. An additional reason for its suitability as a feedstock is its low lignin and high holocellulose content, which, under proper pretreatment conditions, can be solubilized to yield easily fermentable sugars. These are potential sources for the secondary production of high added-value bio-products or biofuels, in a bio-refinery concept [15]. Depending on the pretreatment technique employed, either hemicellulose solubilization—in acid or thermal pretreatments—or lignin degradation—in alkaline or biological ones—might take place [16,17,18]. The main obstacle during pretreatment, especially under severe acidic or alkaline conditions, is the possibility of secondary oxidation reactions taking place, forming inhibitory by-products or toxic compounds [2,17].

Apart from the pretreatment, two other steps are needed for the conversion of a lignocellulosic biomass such as GL to bioethanol or biohydrogen: an enzymatic hydrolysis step, where further saccharification via enzymes takes place, and a fermentation step, where specific microorganisms ferment soluble sugars towards the desirable products. The latter steps can take place either separately (separate hydrolysis and fermentation—SHF) or simultaneously (simultaneous saccharification and fermentation—SSF) [6]. In terms of bioethanol production (BP), although *Saccharomyces cerevisiae* remains the world’s most exploited yeast, its wild strains are in general incapable of fermenting C5 sugars, such as xylose and arabinose, released from hemicellulose degradation [19]. Contrary to *S. cerevisiae*, other yeasts from genera *Pichia*, *Candida* or *Pachysolen* have been proven to be capable of metabolizing C5 sugars towards ethanol [20,21]. However, *S. cerevisiae* has been extensively used to produce ethanol from different kinds of grasses, such as Miscanthus, switch grass, Napier or elephant grass, either in SSF or SHF mode and after different pretreatment conditions [17,22,23]. The use of co-cultures of *S. cerevisiae* and *Trichoderma reesei* or *Aspergillus niger* [24] or genetic modification of wild strains [25] aiming either at higher enzymatic efficiency or at higher ethanologenic capacity is important for developing effective processes for conversion of grasses to bioethanol. Finally, process integration through the use of different concepts, i.e., SSF of untreated napier grass, followed by pentose fermentation [26], is reported as another possibility for higher ethanol concentrations and yields, while fully exploiting the biomass at the same time.

Regarding dark fermentation (DF) of GL via mixed microbial cultures, pretreatment methods such as chemical (alkali or acid), physical (e.g., ultrasound, microwave, ionizing radiation) or a combination of the above are reported to enhance biological hydrogen production (BHP) yields, through rendering grass holocellulose more accessible to fermentative bacteria [11,27,28,29].

Recently, the effect of different pretreatment methods, such as thermal, alkaline (NaOH) and acid (through H_2_SO_4_, H_3_PO_4_ and HCl) at different chemical loadings (2, 10 and 20 g/100 g total solids (TS)) on methane production, through biochemical methane potential (BMP) experiments, was assessed [13]. In that study, different process schemes were developed and compared and the experimental results showed that the methane yield was enhanced with alkaline pretreatment, and the higher the NaOH concentration (20 g/100 g TS), the higher the methane yield observed. In the present study, the effect of the above pretreatment methods of GL waste on BHP, through mixed microbial cultures, and on bioethanol concentrations and yields, through the C5 yeast *Pichia stipitis*, was evaluated in a comparative way, for the first time. Both processes were carried out in SSF mode using the whole pretreatment slurries, during which selection of the optimum pretreatment conditions was performed. The best pretreatment strategies for each process based on biofuels yields (H_2_SO_4_ and NaOH 10 g/100 g TS for BHP and HCl 2 g/100 g TS as well as H_2_SO_4_ and H_3_PO_4_ 10 g/100 g TS for bioethanol production (BP)), were further evaluated under different process schemes aiming at maximizing the product yields and the extent of GL exploitation. Specifically, for BHP, process schemes such as a) fermentation without enzymes addition, b) hydrolysis at a separate step, at SHF and c) separation of the whole pretreatment slurry and use of the separate fractions, were also assessed. For BP, other promising ethanol-producing yeasts such as *Pachysolen tannophilus* or the traditional glucose-fermenting yeast *S. cerevisiae* were also tested, in both SSF or SHF mode, using either the whole pretreatment slurry or the separate fractions obtained after pretreatment. Combinations of the yeasts for selective use at each fraction (i.e., *P. stipitis* and *P. tannophilus* for the rich in C5: sugars hydrolysates and *S. cerevisiae* for the rich in cellulose solid fractions in SSF or SHF) was also performed, aiming at enhanced ethanol yields. To our knowledge, it is the first comparative study, simultaneously investigating hydrogen and bioethanol from GL and evaluating all the possible process schemes, in order to achieve, if possible, the maximum recoverable energy in the form of gaseous and liquid biofuels.

## 2. Results and Discussion 

### 2.1. Composition of GL before and after Pretreatments 

The composition of the GL used is presented in Table 1, where it is obvious that holocellulose, which is the main carbon source for fermentations, accounts for 44.4% of the total dry weight. In Table 2, the effect of all pretreatment methods on each lignocellulosic fraction, is presented. As also confirmed by other studies [16,17], all acid pretreatment methods applied in the present study resulted in hemicellulose solubilization, with 20 g/100 g TS HCl or H_2_SO_4_ leading to a 93.4% and 77.9% reduction, respectively, while NaOH pretreatment was more effective in lignin breakdown, with 10 and 20 g NaOH/100 g TS causing a 61.7% and 94.5% lignin removal, respectively.

**Figure 1:**
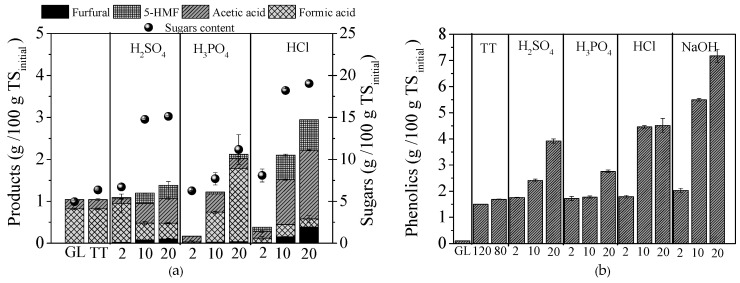
(**a**) Products (furfural, 5-HMF, acetic and formic acid) released and sugars content during thermal treatment (TT) at 120 °C (1 h) and acid (H_2_SO_4_, H_3_PO_4_ and HCl) pretreatment methods at 2, 10 and 20 g/100 g TS and (**b**) phenolic compounds during TT at 120 °C (1 h) or 80 °C (24 h), acid and alkali (NaOH) pretreatment methods tested. Concentrations of products and phenolic compounds of untreated GL waste is also presented.

Cellulose and hemicellulose degradation results in their solubilization towards the respective monosaccharides or oligosaccharides, which are counting in the overall concentration of soluble sugars, presented in Figure 1a. Thus, for H_2_SO_4_ at a concentration of 10 and 20 g/100 g TS, the sugars content was 14.8 ± 0.1 and 15.1 ± 0.1 g/100 g TS, while for HCl it was 18.2 ± 0.7 and 19.0 ± 0.6 g/100 g TS, respectively.

During pretreatment at high temperatures and low pH values, such as acid, a part of the sugars could be degraded into other compounds, such as furanic or furaldehydes and formic acid, which are reported as inhibitors to the yeast or bacteria which are implicated in the subsequent bioprocess [30,31,32]. Xylose degradation results to the production of furfural, while 5-hydroxymethylfurfural (5-HMF) comes from glucose oxidation [31]. Formic acid can be produced either from further oxidation of 5-HMF or from furfural breakdown [17]. Acetic acid is another product released during pretreatment as a result of hemicellulose bond cleavage, while phenolic compounds are formed from the degradation of lignin, which mainly occurs in alkaline pretreatments [16,17,31]. In Figure 1a, the concentrations of furaldehydes, acetic and formic acids, which are produced during the acid pretreatment methods used, are presented, while in Figure 1b, production of the phenolic compounds due to all pretreatments is depicted. From Figure 1a, it is obvious that formation of inhibitors increased with the increased chemical loadings of the acids, and thus, the higher concentrations of H_2_SO_4_ or HCl led to higher production of furaldehydes and acids; on the other hand, higher NaOH concentrations resulted in higher release of phenolic compounds. Specifically, for 20 g HCl/100 g TS, the production of 0.38 ± 0.00 g/100 g TS (0.19 ± 0.00 g/L) furfural and 0.73 ± 0.00 g/100 g TS (0.36 ± 0.00 g/L) 5-HMF was observed, while for 20 g NaOH/100 g TS, the phenolic compounds were 7.18 ± 0.25 g/100 g TS (the respective concentration for 2 g NaOH/100 g TS was 2.51 ± 0.05 g/100 g TS).

### 2.2. BHP Experiments

#### 2.2.1. BHP under Different Enzymatic Loadings

Preliminary BHP experiments were performed under different enzymatic loadings (0, 15, 40 and 100 FPU/g TS GL of cellulase, while glycosidase increased proportionally to the cellulase loading (3:1 (*v*/*v*)) so as to determine the optimum concentration of the enzymatic mixture. The results showed that the yield of BHP from GL without enzyme addition was only 11.6 ± 1.2 mL H_2_/g TS_initial_ (where TS_initial_ are the TS of the initial GL biomass) and increased to 46.9 ± 0.0, 81.8 ± 2.3 and 182.6 ± 10.7 mL H_2_/g TS_initial_, when 15, 40 and 100 FPU cellulase/g TS and glycosidase, at 3:1 (*v*/*v*), were added. The fact that the increase of enzyme loading from 40 to 100 FPU/g TS (2.5 times) did not cause the anticipated increase in hydrogen yield indicated that the lower concentration might be preferable from an economic point of view, due to the high cost of the commercial enzymes. Thus, the concentration of 40 FPU/g TS of cellulase was used in the BHP experiments in SSF and SHF modes. However, a detailed techno-economic evaluation is required for the final selection decision, taking into account not only the process economics but also technical aspects such as the hydrogen production yields.

#### 2.2.2. BHP of the Whole Pretreated Slurry, at SSF

**Figure 2:**
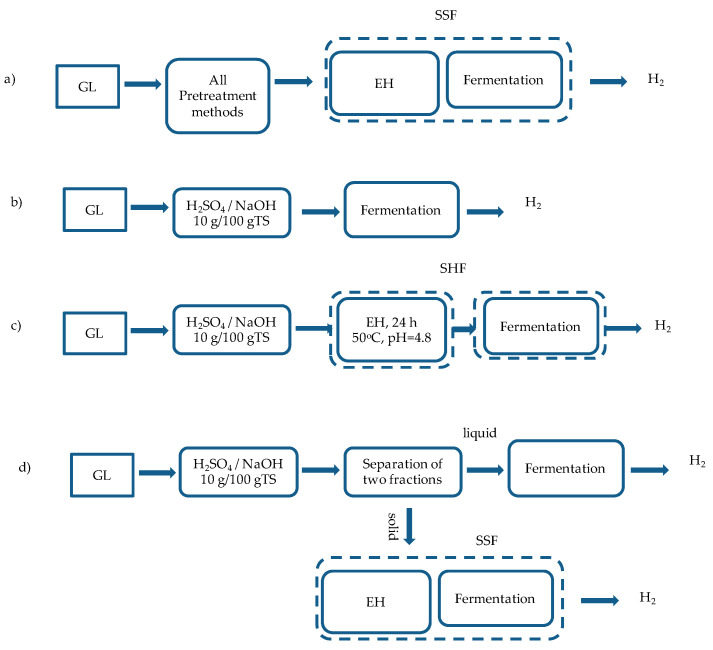
Different process schemes which were evaluated for biological hydrogen production (ΒHP) of grass lawn (GL) (EH: enzymatic hydrolysis): (**a**) the whole slurry of all thermo-chemical pretreatments was used for BHP in SSF (simultaneous saccharification and fermentation) mode. The whole slurry of pretreatment with H_2_SO_4_ and NaOH, 10 g/100 g TS was used (**b**) directly for BHP without addition of enzymes, (**c**) or in a separate hydrolysis and fermentation (SHF) scheme (**d**) or separated and the liquid fraction was used for BHP without enzyme addition, whereas the solid fraction was used in an SSF concept.

BHP experiments were carried out at raw and thermo-chemically pretreated samples, which were obtained at different process schemes (Figure 2). Firstly, the effect of all thermo-chemical pretreatments on BHP from GL waste was investigated using the whole pretreatment slurry and 40 FPU cellulase/g TS (and glycosidase at a ratio 3:1 (*v*/*v*)), in an SSF concept (Figure 2a).

**Figure 3:**
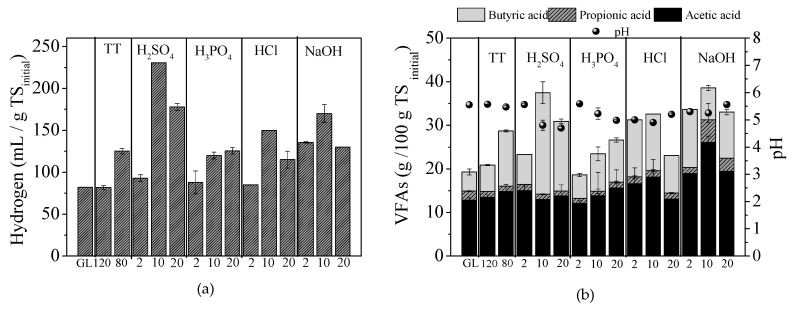
Effect of thermal (TT) at 120 °C (1 h) or 80 °C (24 h), acid (H_2_SO_4_, H_3_PO_4_ and HCl) and alkali (NaOH) pretreatment on (**a**) hydrogen yields and (**b**) volatile fatty acids (VFAs—acetic, propionic and butyric acid) produced in biological hydrogen (BHP) experiments using the whole pretreatment slurry, at simultaneous saccharification and fermentation (SSF).

In Figure 3a, the hydrogen yield of untreated GL and that of thermally treated (80 or 120 °C) or pretreated with acids (H_2_SO_4_, H_3_PO_4_ and HCl) and NaOH at the concentrations of 2, 10 and 20 g/100 g TS, are presented, while pH and the main volatile fatty acids (VFAs) produced (acetic, propionic, butyric acid), at the end of BHP experiments, are presented in Figure 3b. It is obvious that almost all pretreatment methods applied in this study enhanced hydrogen production from GL, under SSF. Pretreatment with 10 g H_2_SO_4_/100 g TS led to the highest BHP yield, which was 230.7 ± 1.6 mL/g TS_initial_ or 276.8 mL/g VS_initial_ (VS_initial_: volatile solids of initial biomass) of GL. This yield was 2.82 times higher than the respective one of raw GL (untreated) under SSF using 40 FPU cellulase/g TS and 20 times higher than for raw GL without enzymes addition. Pretreatments with 20 g H_2_SO_4_/100 g TS and 10 g NaOH /100 g TS also exhibited high BHP yields, which were 177.9 ± 4.1 mL/g TS_initial_ and 170.0 ± 10.7 mL/g TS_initial_, respectively. HCl, NaOH and H_2_SO_4_, at a concentration of 10 g/100 g TS, led to higher BHP yields compared to the other concentrations (2 and 20 g/100 g TS for each chemical agent). In addition, the BHP of thermal treatment at 80 °C for 24 h was higher than the respective at 120 °C for 1 h, indicating that the higher treatment time had a positive effect on hydrogen production. It should be mentioned that, despite the fact that the sugars content after pretreatment with HCl at concentrations of 10 or 20 g/100 g TS were higher compared to those of 10 H_2_SO_4_ g/100 g TS, the corresponding BHP yields were lower for both cases. This could be attributed to the higher concentrations of 5-HMF and furfural, as well as of formic and acetic acids, which were released (Figure 1a) under the latter conditions. Although furans were also released in the whole pretreatment slurry for 10 g H_2_SO_4_/100 g TS (0.08 ± 0.00 g/100 g TS furfural and 0.24 ± 0.00 g/100 g TS 5-HMF), their presence did not influence the BHP (which was the highest), implying that mixed acidogenic cultures might tolerate these compounds up to this level, in agreement with other studies [2,33].

BHP yields of pretreated GL obtained in the present study are much higher than in other studies. For example, Cui and Shen [11] reported a maximum cumulative yield of 72.2 mL/g TS for grass pretreated with HCl 4%, which was 16.45-fold greater than that from the raw substrate. Similarly, Yang and Wang [28] reported that the BHP of grass increased from 26 mL/g to 32, 53 and 68 mL/g, respectively, after pretreatment with ionizing radiation, acid and a combination of the above. Furthermore, the combination of ultrasound with acid pretreatment led to 84.4 mL/g GL [27]. The high differences in BHP yields could be attributed to the extra step of hydrolysis via commercial enzymes, which was accomplished together with the fermentation in the present study. The enzymatic hydrolysis was obviously necessary, since mixed microbial acidogenic cultures, properly heat-treated, were unable to hydrolyze/degrade cellulose, which remained in all pretreated substrates at high concentrations (Table 2). The low BHP value of the untreated GL (11.6 ± 1.2 mL H_2_/g TS_initial_) was in line with other studies (4.4–10.2 mL/g TS) [11,27], indicating the low hydrolytic efficiency of the implicated microorganisms, which increased to 81.8 ± 2.2 mL/g TS_initial_ after addition of enzymes at SSF. Hydrogen yields were also calculated based on the available carbohydrates concentration contained in the raw GL (L/g_carb_), which is the sum of holocellulose and soluble sugars (46.8 g/100 g TS) and was found to vary from 0.17 L/g_carb_ (raw GL in SSF) to 0.49 L/g_carb_ (10 g H_2_SO_4_/100 g TS in SSF).

The main metabolic products detected at the end of fermentations were acetic, butyric and propionic acids, while iso-butyric, iso-valeric, n-valeric and caproic acids were not detected at all. Acetate and butyrate were the dominant metabolic products, with the concentration of acetate being higher in almost all experiments, except for 10 and 20 g H_2_SO_4_/100 g TS, where butyrate was the prevailing metabolite. No correlation was found between BHP and acetate (Pearson’s r = 0.256), while a correlation was found with butyrate production (Pearson’s r = 0.789). Butyric acid concentration was the highest for 10 g H_2_SO_4_/100 g TS, where maximum BHP was observed. Under these conditions, the pH at the end of fermentation was 4.8, which was much lower compared to the other values reported in the literature as suitable for BHP [34]. A correlation of butyrate to the BHP has also been reported for the fermentation of other substrates such as sunflower straw biomass [2] or sweet sorghum extract [9,34].

#### 2.2.3. BHP for Different Hydrolysis and Fermentation Concepts 

For GL pretreated with H_2_SO_4_ and NaOH at a concentration of 10 g/100 g TS, which exhibited higher hydrogen yields, BHP experiments were performed under different hydrolysis and fermentation schemes (Figure 2), and the main results are presented in Table 3. Thus, the whole pretreatment slurry was used directly for fermentation in BHP experiments, without the addition of enzymes (Figure 2b), or in an SHF scheme (Figure 2c). In addition, the liquid fraction (hydrolysate) was used for BHP without enzyme addition, whereas the solid fraction was used in an SSF concept (Figure 2d).

It is obvious that when acid-pretreated GL (the whole slurry) (Figure 2b) was directly used for BHP, without addition of enzymes, a slight increase was observed compared to the untreated GL, from 11.6 to 19.2 mL/g TS_initial_, indicating the necessity for enzymes addition. On the contrary, NaOH pretreatment without enzymes addition (Figure 2b) did not enhance the BHP. Similar low BHP yields were reported by Cui and Shen [11], who observed an increase from 4.4 to 16.3 and 19.3 mL/g, when GL waste was pretreated with HCl and NaOH 10 g/100 g TS, for 30 min at 100 °C.

Moreover, the use of enzymes as a separate step (1 day at 50 °C), prior to fermentation (SHF), using the whole slurry, was evaluated (concept Figure 2c). Despite the fact that during SHF, hydrolysis and fermentation were performed at the optimum for each process conditions, for both pretreatment methods, the SSF mode led to higher BHP yields than SHF. This could be attributed to the fact that during hydrolysis, a possible contamination might have occurred, which resulted in a loss of sugars, which could be possibly transformed to hydrogen in the subsequent fermentation step. A sterilization step follows, just before the hydrolysis step could be a solution in order to avoid this possible contamination, achieving higher hydrogen yields. These results are in agreement with Quemeneur et al. [35], who studied the effect of enzymes’ addition on BHP from wheat straw in SSF and SHF, using mixed microbial cultures. They observed a two-fold increase in hydrogen production yields, after enzymatic treatment at SSF compared to the SHF, due to consumption of free sugars by indigenous wheat straw microorganisms during enzymatic hydrolysis.

Finally, the fermentation after separation of the whole pretreatment slurry was assessed (concept Figure 2d) for both pretreatment conditions. In Table 3, the BHP yields for solids are expressed per kg of initial TS, meaning that the material recovery due to the loss of weight during pretreatment has been taken into account. In the case of pretreatment with 10 g H_2_SO_4_/100 g TS, separation of both fractions enhanced BHP yields, since 190.1 ± 16.7 mL/g TS_solids_ (TS_solids_:TS of the pretreated solids) or 111.1 ± 9.8 mL/g TS_initial_ were produced from the solid fraction and 159.1 ± 12.8 mL/g TS_initial_ (based on the calculation that 100 mL of acid solution were mixed with 5 g TS_initial_ and no liquid was lost during separation) from the hydrolysate. Comparing the BHP of the sum of both fractions, expressed in mL/g TS_initial_ with the respective of the whole slurry at each pretreatment method, it is obvious that separation of the pretreated biomass was favored for 10 g H_2_SO_4_/100 g TS (270.2 mL/g TS_initial_, while 230.7 ± 1.6 mL/g TS_initial_ were produced at SSF). This could be attributed to the high BHP of the hydrolysate, which also verifies that the products released during pretreatment did not inhibit fermentative bacteria to produce hydrogen.

### 2.3. BP Experiments

#### 2.3.1. BP of the Whole Pretreatment Slurry, at SSF

**Figure 4:**
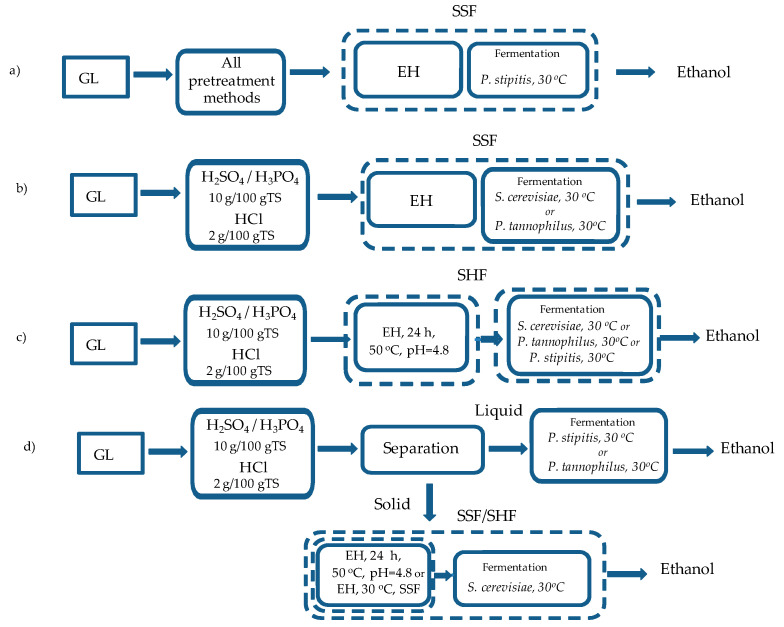
Different process schemes which were evaluated for bioethanol production (BP) of GL (EH: enzymatic hydrolysis). (**a**) the whole slurry of all thermo-chemical pretreatments was used for BP using *Pichia stipitis* in SSF (simultaneous saccharification and fermentation) mode. The whole slurry of pretreatment with H_2_SO_4_ and H_3_PO_4_, 10 g/100 g TS and HCl, 20 g/100 g TS was used for BP (**b**) using *P. stipitis*, *Pachysolen tannophilus* and *Saccharomyces cerevisiae* in SSF (**c**) or at separate hydrolysis and fermentation (SHF), (**d**) or separated and the liquid fraction was used for BP using *P. stipitis*/*P. tannophilus*, whereas the solid fraction was fermented by *S. cerevisiae* in SSF or SHF concept.

**Figure 5:**
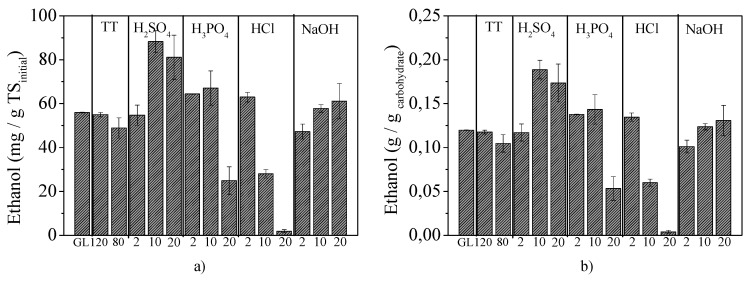
Effect of thermal (TT) at 120 °C (1 h) or 80 °C (24 h), acid (H_2_SO_4_, H_3_PO_4_ and HCl) and alkali (NaOH) pretreatment on ethanol yields expressed as (**a**) mg/g TS_initial_ and (**b**) g/g_carbohydrate_ available, when using the whole pretreatment slurry, at SSF.

BP experiments were performed with raw and thermo-chemically pretreated samples either of the whole pretreatment slurry or of the separated fractions (Figure 4). Initially, *P. stipitis* was used for bioethanol production from GL under SSF using the whole slurry obtained after all pretreatment methods (Figure 4a). The use of *P. stipitis* in these experiments was based on its ability to ferment both C5 and C6 sugars towards ethanol since the slurry coming from acid pretreatments contained high xylose concentrations, due to hemicellulose degradation, as presented in Antonopoulou et al. [13]. In Figure 5, the ethanol production yields, expressed as mg/g TS_initial_ (Figure 5a) or per g of available carbohydrates (the sum of holocellulose and soluble sugars) contained in raw GL (g/g_carbohydrate_) (Figure 5b), are presented. The ethanol efficiency could be compared to the maximum theoretical value of 0.511 g/g_carbohydrates consumed_ (g/g of consumed carbohydrates), taking into account that this yield refers to the g of consumed carbohydrates and not to the initially available. Thus, the values presented in Figure 5b are quite underestimated compared to the theoretical ones, since it was assumed that a 100% carbohydrates consumption had occurred.

Raw GL at SSF produced 56.0 ± 0.1 mg ethanol/g TS_initial_ or 0.12 ± 0.00 g/g _carbohydrate_, which was 23.4% of the maximum theoretical. Acid pretreatment with H_2_SO_4_ resulted in higher ethanol yields, i.e., treatment with 10 and 20 g/100 g TS led to the production of 88.3 ± 5.0 and of 81.2 ± 8.1 mg/g TS_initial_ (or 0.19 ± 0.01 and 0.17 ± 0.02 g/g_carbohydrate_, respectively), corresponding to a 58% and 45% increase compared to the respective of raw GL at SSF. Regarding pretreatment with HCl, ethanol yields decreased with the increase of acid concentration; thus, pretreatment with 2, 10 and 20 g /100 g TS led to the production of 63.0 ± 0.1, 28.1 ± 1.9 and just 1.8 ± 0.7 mg/g TS_initial_, respectively. The low ethanol yields observed could be attributed to a partial or total inhibition of *P. stipitis* due to the high concentration of compounds released during pretreatment. Under the latter conditions, high furfural and 5-HMF as well as high acetic acid concentration were observed (Figure 1a). However, the above concentrations are lower than the respective reported as inhibitory for *P. stipitis* growth, when furans or acetic acid were added separately, in a xylose-rich substrate [36]. It should be mentioned, however, that the simultaneous presence of all the above might have a synergistic toxic effect, causing partial or total inhibition to the same yeast, even though this did not happen when they are added separately [37,38].

Contrary to the results of the present work, in a previous study, where HCl and H_2_SO_4_ were used as pretreatment methods for ethanol production from sunflower straw biomass using *P. stipitis* in an SSF concept, the addition of high H_2_SO_4_ loading (20 g/100 g TS) led to lower ethanol yields, while pretreatment with 20 gHCl/100 g TS led to the optimum ethanol production efficiency, of 104 mg/g TS_initial_ [2]. Demiray et al. [39] applied H_2_SO_4_ pretreatment under different loadings in order to enhance ethanol production form pomegranate peel, under different experimental conditions, comparing the ethanologenic efficiencies of *P. stipitis* and *S. cerevisiae*, under SSF. They observed that at the optimal conditions, *P. stipitis* produced 2.9 g/L ethanol (0.29 g/g), while the respective values for *S. cerevisiae* were 5.6 g/L and 0.43 g/g. In the majority of the studies, *P. stipitis* has been used for ethanol production from the hydrolysates obtained from pretreatment of different kinds of lignocellulosic feedstocks, with promising ethanol production yields [40,41,42].

#### 2.3.2. BP under Different Fermentation Concepts 

Pretreatment with 10 g H_2_SO_4_ or H_3_PO_4_/100 g TS and 2 g HCl/100 g TS, which led to higher ethanol yields using *P. stipitis* at SSF, were tested under different hydrolysis and fermentation schemes, presented in Figure 4.

Apart from *P. stipitis*, *S. cerevisiae* or the C5 yeast *P. tannophilus* were used under SSF (Figure 4b) and also under SHF (Figure 4c). In addition, separation of both fractions was performed, where the rich in xylose hydrolysate was used for ethanol production from *P. stipitis*/*P. tannophilus* at 30 °C (without addition of enzymes), while the rich in cellulose solid fraction was used for ethanol production using *S. cerevisiae* a) in an SSF and b) in an SHF concept (Figure 4d).

**Figure 6:**
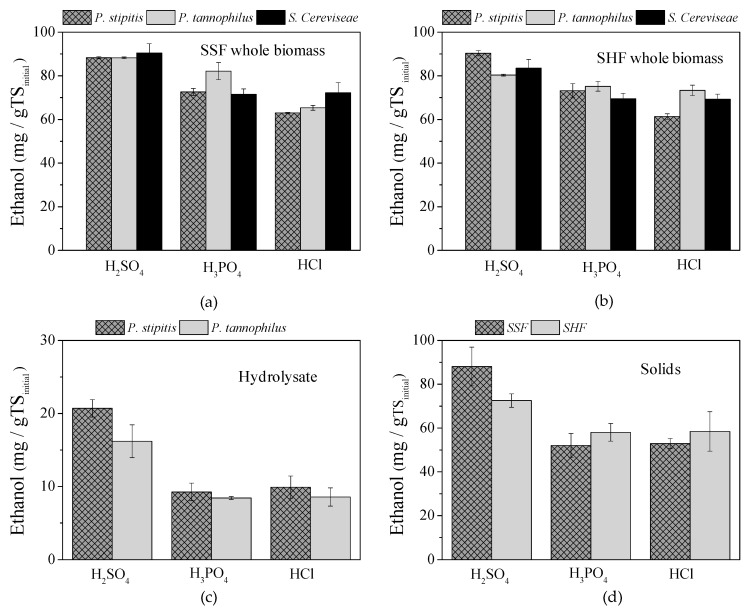
Ethanol yields of GL pretreated with H_2_SO_4_ and H_3_PO_4_ 10 g/100 g TS as well as 2 g HCl/100 g TS under different concepts, i.e., using the whole pretreatment slurry and *P. stipitis*, *P. tannophilus* and *S. cerevisiae* (**a**) at simultaneous saccharification and fermentation (SSF) and (**b**) separate hydrolysis and fermentation (SHF), or after separation of both fractions, where (**c**) the hydrolysate was fermented by *P. stipitis* or *P. tannophilus* and (**d**) the solid fraction by *S. cerevisiae* at SSF or SHF.

In Figure 6a,b, the ethanol yields when using *P. stipitis*, *S. cerevisiae* and *P. tannophilus* in fermentation experiments of the whole pretreatment slurries, under SSF and SHF, are presented. It is obvious that pretreatment with H_2_SO_4_ led to higher ethanol yields and all microorganisms were efficient, either under SSF or SHF (Table 4). In addition, at the same pretreatment conditions, the use of enzymes at a separate step (1 day at 50 °C), prior to fermentation (SHF) did not significantly affect the ethanol yield (*p* > 0.05 in all cases) (i.e., for *P. stipitis*: 88.3 ± 0.5 at SSF and 90.3 ± 1.2 mg/g TS_initial_ at SHF). Similar results were observed for H_3_PO_4_ and HCl pretreatment using *P. stipitis* and *S. cerevisiae*. Using *P. tannophilus* at SSF, the BP was higher than SHF, in case of H_3_PO_4_ and lower, in case of HCl, but the differences were also not significant (*p* > 0.05 in both cases). In Figure 6c,d, the BP yields after separation of the whole pretreatment slurry into liquid and solid fractions are presented. It should be mentioned that the BP yields for solids are expressed per kg of initial TS, implying that the loss of weight during pretreatment (material recovery) has been taken into account. For the hydrolysates, *P. stipitis* slightly enhanced BP compared to *P. tannophilus*; however, the increments were low and no statistical difference was observed (*p* > 0.05 at all cases). López-Abelairas et al. [20] found that *P. tannophilus* led to the highest overall ethanol yield compared to *P. stipitis*, *S. cerevisiae*, *Kluyveromyces marxianus* and *Candida shehatae* when fermenting hydrolysates from biologically pretreated wheat straw, a substrate that was characterized by high hemicellulose content and no inhibitors. For H_3_PO_4_ and HCl, solids fermentation by *S. cerevisiae* was not significantly affected by the hydrolysis step (SSF or SHF) (*p* > 0.05), while for H_2_SO_4_ the SSF concept led to much higher ethanol yields (*p* = 0.144). In the case of H_2_SO_4_, when using *P. stipitis* for the fermentation of the hydrolysate and SSF concept for the pretreated solids, the highest ethanol yield was observed (108.8 mg/g TS_initial_ or 0.24 g/g_carbohydrate_) (47% of the theoretical). Comparing the ΒP of the sum of both fractions with the respective of the whole slurry at each pretreatment method, the separation of the pretreated biomass was favored only for 10 g H_2_SO_4_/100 g TS.

### 2.4. Energy Recovery from Different Process Schemes

Based on the above, it is obvious that the use of the separated fractions versus the whole biomass slurry can be either beneficial, negative or neutral, based on the hydrogen and ethanol production yields. Regardless of the process scheme, pretreatment with 10 g H_2_SO_4_/100 g TS was the optimum for both BHP and BP. Taking into account the maximum yield of each biofuel, the maximum recoverable energy from grass could be estimated, based on the energy densities of hydrogen and ethanol [43]. In terms of hydrogen production, comparing the SSF and SHF concept, the SSF was favorable, not only for the higher yield, but also for the need for one vessel for hydrolysis and fermentation. Based on this, the SSF concept should be compared with the concept of separation of both fractions and fermentation of liquid and solids in different vessels. From an economical point of view, the additional cost for the separation of the two fractions and the need for separate fermentations should be taken into account.

Regarding ethanol production using the whole slurry, *S. serevisiae* in an SSF and *P. stipitis* in an SHF led to similar ethanol yields. Taking into account the aforementioned benefits of SSF versus SHF, the first concept was preferable. Regarding the separation of both fractions, the use of *P. stipitis* for the hydrolysate and that of *S. serevisiae* for the solid fraction at SSF exhibited higher ethanol production and selected as the optimum (Table 5). For comparison, the recoverable energy in the form of methane produced from GL pretreated with 20 g NaOH/100 g TS (whole biomass: 413.5 mL CH_4_/g VS_initial_ or 344.9 mL CH_4_/g TS_initial_ and after separation of both fractions and anaerobic digestion of the separated ones: 427.1 mL CH_4_/g VS_initial_ or 356.2 mL CH_4_/g TS_initial_) [13] is also presented in Table 5.

Regarding fermentations, the energy gained in the form of hydrogen is higher than that in the form of ethanol. In addition, the fact that hydrogen is produced via mixed microbial cultures, without sterilization requirements, as in the case of ethanol, renders it more preferable from an economic point of view. However, the difficulties of hydrogen storage should also be taken into account. Anaerobic digestion for biogas production seems to lead to the highest energy recovery, so that this seems to be the optimum scenario for GL exploitation, in terms of the energy gained. However, the final selection of the product and subsequently on the process scheme will depend not only on the energy yield but also on the end use. For instance, if a liquid transportation fuel is needed, ethanol will be advantageous over gaseous biofuels, even with lower energy yields.

## 3. Materials and Methods

### 3.1. Biomass Used

GL was collected from gardens of the region of Attica, Greece. The fresh biomass was air dried, chopped and milled with a lab grinder (IKA A11 basic) and the powder was sieved (pore size of 0.7 mm) and then air-dried at ambient temperature.

### 3.2. Pretreatment Methods Used

In the present study, the pretreatment methods presented in Antopopoulou et al. [13] were used. Briefly, H_2_SO_4_, H_3_PO_4_ and HCl at 121 °C for 1 h and NaOH at 80 °C for 24 h, at concentrations of 2, 10 and 20 g/100 g TS and a solids loading of 5% (*w*/*v*) were tested. For comparison, thermal treatment (121 °C for 1 h or 80 °C for 24 h) without any chemical addition, was conducted. Depending on the process scheme that was followed, either the whole pretreatment slurry or the two fractions obtained after separation (liquid and solid fractions), through filtering with 0.7 μm, were used.

### 3.3. BHP Experiments

#### 3.3.1. Microbial Cultures 

Anaerobic sludge (pH = 7.6 ± 0.1, total suspended solids (TSS) = 15.7 ± 0.1 g/L and volatile suspended solids (VSS) = 9.8 ± 0.3 g/L) obtained from the wastewater treatment plant digester of the city of Patras (Western Greece), operating at steady state at a hydraulic retention time of 15 d, was used as inoculum. The sludge was initially gassed and boiled at 100 °C for 15 min, as presented in Antonopoulou et al. [2].

#### 3.3.2. BHP Experiments 

BHP experiments were carried out in duplicate at 35 °C, in 160 mL serum bottles. During preliminary experiments, the optimum enzymatic loading was determined in SSF mode, with the addition of Celluclast 1.5 L (Cellulase from *Trichoderma reesei*, ATCC 26921) at concentrations of 0, 15, 40 and 100 FPU/g TS and Novozyme 188 (Cellobiase from *Aspergillus niger*) at a ratio of 3:1 (*v*/*v*). These experiments were performed as proposed in Antonopoulou et al. [2].

First, the whole pretreatment slurry obtained after all chemical and thermal pretreatment methods tested was used for BHP experiments in which the hydrogen yield for a period of 72 h was assessed, through SSF (Figure 2a). Based on the experimental results of these tests, H_2_SO_4_ and NaOH at a concentration of 10 g/100 g TS, were applied under different hydrolysis and fermentation schemes: the whole pretreatment slurry was used a) directly for fermentation in BHP experiments for a period of 72 h without the addition of enzymes (Figure 2b) and b) for enzymatic hydrolysis for 1 d at 50 °C (which is the optimum temperature for the enzymes) and pH 5 (through citrate buffer 1M) and then for BHP in 35 °C for 72 h (Figure 2c) in SHF. Finally, the whole pretreatment slurry was separated in two fractions, where the liquid fraction was used for BHP without enzymes addition, whereas the solid fraction was used for BHP experiments in SSF for 72 h (Figure 2d).

For the experiments with the whole slurry, the procedure described in Antonopoulou et al. [2] was followed. For the solid fractions obtained after pretreatment, 10 mL of mixed anaerobic culture, 30 mL of basal nutrient medium (containing NaH_2_PO_4_*2H_2_O: 8.98 g/L, Na_2_HPO_4_*2H_2_O: 5.2 g/L, yeast extract: 0.625 g/L), 10 mL/L of the solution of trace elements [3] and 10 mL of water were mixed with appropriate amounts of pretreated solids so as the final TS being 1%. Finally, for the experiments with the liquid fractions, 10 mL of the hydrolysates were used, along with the inoculum (10 mL), the nutrient basal medium (30 mL) and the solution of the trace elements. Addition of 40 FPU/g TS GL of Celluclast 1.5 L and Novozyme 188 at a ratio of 3:1 (*v*/*v*) was performed either for SSF or for SHF. The pH, temperature and agitation were monitored according to Antonopoulou et al. [2]. Blank experiments were also carried out, containing only the inoculum, while the content of the vials was gassed with inert gas, in order to secure anaerobic conditions. At the end of the experiments, the pH was measured and the liquid content was centrifuged, which was filtered through 0.7 μm filters and analyzed for VFAs production.

### 3.4. BP Experiments

#### 3.4.1. Cultures and Media

*P. stipitis* CECT 1922, *S. cerevisiae* CECT 1332 and *P. tannophilus* CECT 1426 were obtained by Spanish type culture collection (CECT), as freeze-dried cultures. *P. stipitis* and *S. cerevisiae* were maintained in a medium (CECT number 138) of the following composition: 10 g/L glucose, 5 g/L mycopeptone, 3 g/L yeast extract and 3 g/L malt extract, while *P. tannophilus* was maintained in a medium (CECT number 63) containing: 20 g/L glucose, 1 g/L mycopeptone and 20 g/L malt extract. The flasks were incubated at 30 °C and shaken at 150 rpm for 20 h. Stock cultures were stored at −80 °C in 20% glycerol and inoculation cultures were transferred twice before use.

#### 3.4.2. Fermentation/Hydrolysis Experiments

Fermentation experiments were carried out in duplicate, in serum vials of 20 mL, under micro-aerobic conditions. Cells from pre-cultures of the above microorganisms, in the late exponential phase, at an initial concentration of 0.8 g/L, were centrifuged at 4500× *g* for 15 min and the yeast pellets were re-suspended in a mineral solution containing KH_2_PO_4_, MgCl_2_·6H_2_O and (NH_4_)_2_SO_4_, each at a concentration of 1 g/L.

BP experiments were performed with raw and pretreated samples either of the whole pretreatment slurry or of the separated fractions (Figure 4). In the first case, 15 mL of the whole slurry at a solids load of 5% TS (*w*/*v*) was used, while in the second case, either 15 mL of the hydrolysates or 15 mL of aquatic solutions of the pretreated solids (5% TS) was used. In addition, 45 FPU/g TS GL of Celluclast 1.5 L and Novozyme 188 at a ratio of 3:1 (*v*/*v*) were used, either in SSF or SHF. Initially, the whole pretreatment slurry from all thermo-chemical pretreatment methods was used for BP through SSF at 30 °C, for 50 h using the C5 yeast *P. stipitis* (Figure 5a). Based on the experimental results of these tests, H_2_SO_4_ and H_3_PO_4_ at a loading of 10 g/100 g TS and 2 g/100 g TS HCl found to be the optimum and thus used for BP at SSF either using *S. cerevisiae* or the C5 yeast *P. tannophilus* at 30 °C, for 50 h (Figure 4b), or at SHF, where prior to fermentation at 30 °C, enzymatic hydrolysis of the sterilized pretreated GL was carried out for 24 h at 50 °C and pH 4.8 (Figure 4c). In addition, separation of both fractions was performed, where the rich in xylose hydrolysate was used for ethanol production from *P. stipitis*/*P. tannophilus* at 30 °C (without the addition of enzymes), while the rich in cellulose solid fraction was used for ethanol production using *S. cerevisiae* a) in a SSF and b) in a SHF concept (Figure 4d). Addition of 45 FPU/g TS GL of Celluclast 1.5 L and Novozyme 188 at a ratio of 3:1 (*v*/*v*) was performed either in SSF or in SHF. The experimental procedure described in Antonopoulou et al. [2] was followed.

### 3.5. Analytical Methods 

The procedure followed for samples characterization before and after pretreatment in terms of their lignocellulosic content (extractives, cellulose, hemicellulose and lignin) is described in Antonopoulou et al. [2,13]. The liquid fractions were characterized in terms of their soluble carbohydrates’ content, phenolic compounds as well as furaldehydes (5-HMF and furfural) and aliphatic acids (formic and acetic acid) as described in Antonopoulou et al. [2,17].

The measurements of TS, VS, TSS, VSS and Τotal Kjeldahl Νitrogen (TKN) were performed according to Standard Methods [44], where the crude protein content was determined by multiplying TKN by a factor of 6.25 [13].

The filter paper activity (FPU) of cellulase (Celluclast 1.5 L), the hydrogen content of the produced gas as well as the ethanol and the VFAs (acetic, propionic, iso-butyric, butyric, iso-valeric, valeric, hexanoic) concentrations were quantified as described in Antonopoulou et al. [2].

### 3.6. Statistical Analysis 

A two-sample t-test with a threshold *p*-value of 0.05 was applied in order to analyze statistically the effect of pretreatment and fermentation parameters on BP of GL.

## 4. Conclusions

The experimental results obtained showed that pretreatment of GL waste with 10 g H_2_SO_4_/100 g TS led to the highest BHP and BP yields, especially when the two fractions obtained after pretreatment were fermented separately (270.1 mL H_2_/g TS_initial_ correlating to 3.4 MJ/kg TS and 108.8 mg ethanol/g TS_initial_ correlating to 2.9 MJ/kg TS). From an economic point of view, the additional cost for the separation of the two fractions and the need for separate fermentations should be taken into account and compared with the concept of using the whole pretreated slurry, at SSF (230.7 mL H_2_/g TS_initial_ correlating to 2.9 MJ/kg TS_initial_ and 90.5 mg ethanol/g TS_initial_ correlating to 2.4 MJ/kg TS_initial_).

## Figures and Tables

**Table 1:** The main characteristics of grass lawn (GL) used in the present study. TS: total solids; VS: volatile solids.

Characteristic	Value
TS (%)	92.2 ± 0.1
VS (g/100 g TS)	83.4 ± 0.1
Cellulose (g/100 g TS)	20.4 ± 0.1
Hemicellulose (g/100 g TS)	24.0 ± 2.0
Lignin (g/100 g TS)	12.3 ± 1.2
Acid Insoluble lignin	7.6 ± 0.4
Soluble lignin	4.7 ± 0.1
Extractives (g/100 g TS)	25.6 ± 3.1
Proteins (g/100 g TS)	10.5 ± 0.5

**Table 2:** Effect of pretreatment on the lignocellulosic fraction of GL [13].

Pretreatment Conditions		Reduction (%)		
		Lignin	Cellulose	Hemicellulose
Thermal	80 °C	0.0 ± 0.0	0.0 ± 0.0	1.7 ± 0.2
	120 °C	0.0 ± 0.0	0.9 ± 0.1	0.8 ± 0.1
H_2_SO_4_	2 g/100 g TS	0.0 ± 0.0	0.8 ± 0.0	4.7 ± 0.4
	10 g/100 g TS	0.0 ± 0.0	1.1 ± 0.2	54.1 ± 0.8
	20 g/100 g TS	0.0 ± 0.0	3.4 ± 0.4	77.9 ± 1.4
H_3_PO_4_	2 g/100 g TS	0.0 ± 0.0	3.5 ± 0.4	7.3 ± 0.3
	10 g/100 g TS	0.0 ± 0.0	4.5 ± 0.2	14.6 ± 0.5
	20 g/100 g TS	0.0 ± 0.0	5.1 ± 0.7	33.8 ± 1.4
HCl	2 g/100 g TS	3.3 ± 0.3	0.0 ± 0.0	9.2 ± 0.5
	10 g/100 g TS	6.6 ± 0.4	0.0 ± 0.0	84.4 ± 1.2
	20 g/100 g TS	6.8 ± 0.5	0.1 ± 0.0	93.4 ± 2.4
NaOH	2 g/100 g TS	16.7 ± 1.5	7.1 ± 0.4	10.3 ± 1.5
	10 g/100 g TS	61.7 ± 1.2	6.8 ± 0.2	23.5 ± 0.3
	20 g/100 g TS	94.5 ± 1.4	5.4 ± 0.8	31.8 ± 0.5

**Table 3:** Hydrogen yields (mL/g TS_initial_) of all schemes applied in this study for GL pretreated with H_2_SO_4_ and NaOH, 10 g/100 g TS. SSF: simultaneous saccharification and fermentation, SHF: separate hydrolysis and fermentation.

Whole Biomass Slurry				Separated Fractions		
	No Enzymes ^a^	SSF ^b^	SHF ^c^	Liquid ^d^	Solid-SSF ^d^	Sum
H_2_SO_4_	19.2 ± 0.3	230.7 ± 1.6	154.5 ± 18.8 ^c^	159.1 ± 12.8	111.1 ± 9.8	270.2
NaOH	11.2 ± 0.7	170.0 ± 10.7	100.6 ± 6.6 ^c^	85.7 ± 12.7	85.0 ± 6.7	170.7
Untreated	11.6 ± 1.2	81.8 ± 2.2	65.3 ± 5.4 ^c^	n.t. ^e^	n.t. ^e^	

^a^ Concept Figure 2b, ^b^ Concept Figure 2a, ^c^ Concept Figure 2c, ^d^ Concept Figure 2d, ^e^ n.t.: not tested.

**Table 4:** Ethanol yields (mg/g TS_initial_) of all schemes applied in this study for GL pretreated with H_2_SO_4_ and H_3_PO_4_ 10 g/100 g TS and 2 g HCl/100 g TS. SSF: simultaneous saccharification and fermentation, SHF: separate hydrolysis and fermentation.

Whole Biomass Slurry							Separated Fractions			
	*P. stipitis*		*P. tannophilus*		*S. cerevisiae*		*P. stipitis (Liquid)*		*P. tannophilus (Liquid)*	
	SSF	SHF	SSF	SHF	SSF	SHF	Solid SSF ^a^	Solid SHF ^a^	Solid SSF ^a^	Solid SHF ^a^
H_2_SO_4_	88.3 ± 0.5	90.3 ± 1.2	88.2 ± 0.4	80.3 ± 0.4	90.5 ± 4.2	83.5 ± 4.1	108.8 ± 8.6	92.6 ± 4.3	104.3 ± 9.6	88.8 ± 7.2
H_3_PO_4_	72.6 ± 1.6	73.1 ± 3.3	82.1 ± 3.9	75.2 ± 2.2	71.5 ± 2.5	69.5 ± 2.5	61.3 ± 4.2	67.3 ± 3.9	60.5 ± 2.5	66.5 ± 2.8
HCl	63.0 ± 0.2	61.3 ± 1.2	65.3 ± 1.1	73.3 ± 2.4	72.3 ± 4.6	69.3 ± 2.3	62.8 ± 3.9	68.4 ± 5.9	61.5 ± 3.7	67.0 ± 5.4

^a^ The solid fraction was fermented by *S. cerevisiae* either at SSF or at SHF mode.

**Table 5:** Recoverable energy (MJ/kg TS_initial_) of the best schemes applied in this study for GL, also compared with study [13].

Scheme	Pretreatment, Biofuel, Conditions	Energy (MJ/kg TS)	Source
Whole biomass	10 g/100 g TS H_2_SO_4_, H_2,_ SSF	2.9	This study
	10 g/100 g TS H_2_SO_4_, Ethanol, SSF (*S. cerevisiae*)	2.4	This study
	20 g/100 g TS NaOH, CH_4_	13.7	[13]
Separation of fractions	10 g/100 g TS H_2_SO_4_, H_2_	3.4	This study
	10 g/100 g TS H_2_SO_4_, Ethanol (*P. stipitis/solids—SSF*)	2.9	This study
	20 g/100 g TS NaOH, CH_4_	14.1	[13]
